# Differential roles of caspase-1 and caspase-11 in infection and inflammation

**DOI:** 10.1038/srep45126

**Published:** 2017-03-27

**Authors:** Si Ming Man, Rajendra Karki, Benoit Briard, Amanda Burton, Sebastien Gingras, Stephane Pelletier, Thirumala-Devi Kanneganti

**Affiliations:** 1 Department of Immunology, St. Jude Children’s Research Hospital, Memphis, TN, USA

## Abstract

Caspase-1, also known as interleukin-1β (IL-1β)-converting enzyme (ICE), regulates antimicrobial host defense, tissue repair, tumorigenesis, metabolism and membrane biogenesis. On activation within an inflammasome complex, caspase-1 induces pyroptosis and converts pro-IL-1β and pro-IL-18 into their biologically active forms. “ICE^−/−^” or “*Casp1*^−/−^” mice generated using 129 embryonic stem cells carry a 129-associated inactivating passenger mutation on the *caspase-11* locus, essentially making them deficient in both caspase-1 and caspase-11. The overlapping and unique functions of caspase-1 and caspase-11 are difficult to unravel without additional genetic tools. Here, we generated caspase-1–deficient mouse (*Casp1*^Null^) on the C57BL/6 J background that expressed caspase-11. *Casp1*^Null^ cells did not release IL-1β and IL-18 in response to NLRC4 activators *Salmonella* Typhimurium and flagellin, canonical or non-canonical NLRP3 activators LPS and ATP, *Escherichia coli, Citrobacter rodentium* and transfection of LPS, AIM2 activators *Francisella novicida*, mouse cytomegalovirus and DNA, and the infectious agents *Listeria monocytogenes* and *Aspergillus fumigatus*. We further demonstrated that caspase-1 and caspase-11 differentially contributed to the host defense against *A. fumigatus* infection and to endotoxemia.

Inflammatory caspases, include caspase-1 (human and mouse), caspase-4 (human), caspase-5 (human) and caspase-11 (mouse), contribute to a variety of biological functions^1^,^2^. Caspase-1, also known as interleukin-1β (IL-1β)-converting enzyme or ICE^2^,^3^,^4^,^5^, can directly cleave the precursor cytokines pro-IL-1β and pro-IL-18 at the sites Asp116–Ala117^3^,^4^,^6^,^7^ and Asp35–Asn36^5^,^8^,^9^,^10^, respectively, generating a biologically active form of the cytokines for release by the cell. Characterization of the purified caspase-1 protein in 1992 revealed a heterodimeric cysteine protease composed of two subunits, p10 and p20^11^.

Caspase-1 is activated within an inflammasome complex^12^, a macromolecular protein complex formed in the cytoplasm of a cell on recognition of pathogen-associated molecular patterns and danger-associated molecular patterns by a NOD, LRR-containing protein (NLR), AIM2-like receptor (ALR) or pyrin^13^. In 1995, two independent groups each generated a mouse line lacking the gene encoding caspase-1, called “ICE^−/−^” or “*Casp1*^−/−^”, using embryonic stem cells obtained from the 129 mouse strain^14^,^15^. A later study revealed that these mouse lines lack caspase-11 expression due to a 129-associated passenger mutation on the *caspase-11* locus that potentially mediated rapid decay of the *caspase-11* mRNA^16^. The close proximity of the *caspase-1* and *caspase-11* loci prevented their segregation despite extensive backcrossing to the C57BL/6 background^16^. We will follow the existing convention of the published literature and call previously-generated “ICE^−/−^” and “*Casp1*^−/−^” strains as *Casp1*^−/−^*Casp11*^−/−^ (also known as *Casp1*^−/−^*Casp11*^129mt/129mt^) mice^16^. The biological insights into caspase-1 gained from the use of *Casp1*^−/−^*Casp11*^−/−^ mouse strains should be revisited.

Here, we generated caspase-1–deficient mouse strain on the C57BL/6 J background, referred to as *Casp1*^Null^, to overcome the confounding loss of caspase-11 in existing mouse strains. Bone marrow-derived macrophages (BMDMs) or dendritic cells (BMDCs) from *Casp1*^Null^ mice expressed caspase-11 proteins and did not secrete IL-1β and IL-18 or undergo pyroptosis in response to canonical NLRP3, NLRC4 or AIM2 inflammasome activators. In addition, *Casp1*^Null^ BMDMs failed to secrete IL-1β and IL-18, but underwent caspase-11–dependent pyroptosis, in response to non-canonical activation of the inflammasome. Further, both caspase-1 and caspase-11 contributed to the host defense against infection with *A. fumigatus*, whereas caspase-11 predominantly contributed to lethality during LPS-induced endotoxemia. This caspase-1–deficient mouse strain provides the scientific community with an exciting opportunity to refine the roles of caspase-1 and caspase-11 in health and disease.

## Results

### The *Casp1* ^Null^ mouse line expresses caspase-11

**Figure 1:**
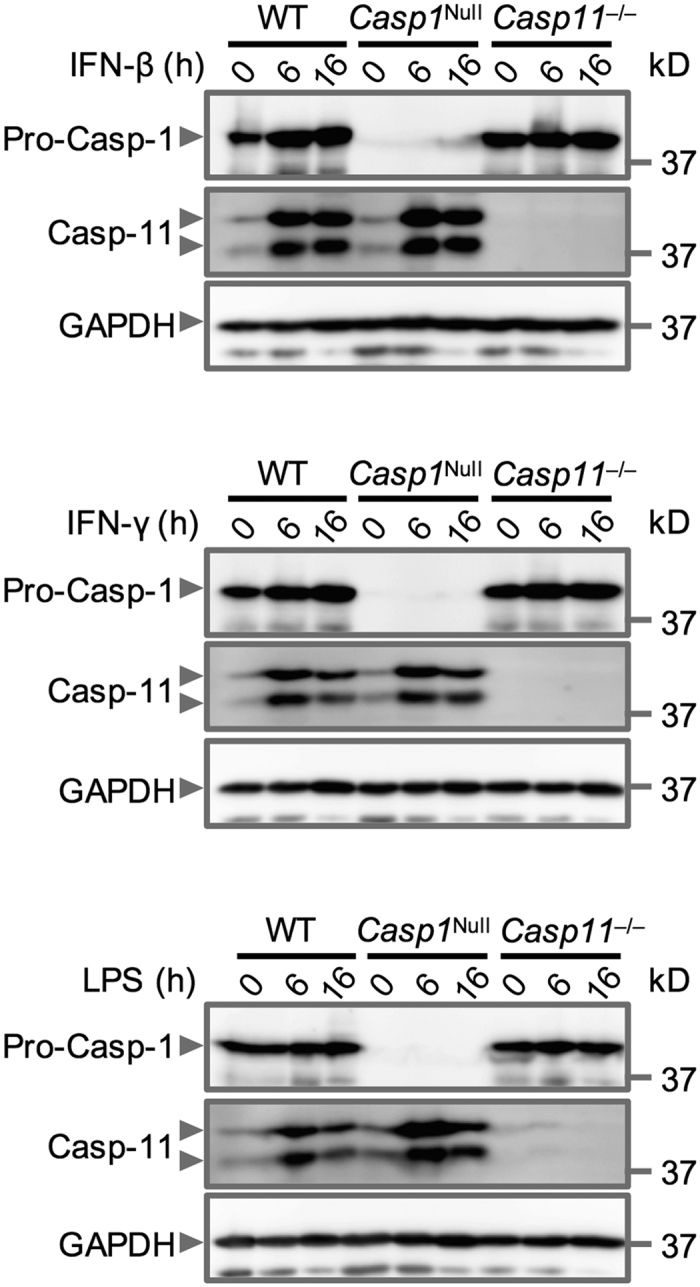
Caspase-11 is expressed in *Casp1*^Null^ bone marrow-derived macrophages. Immunoblot analysis of caspase-1, caspase-11 and GAPDH (loading control) in unprimed WT or mutant BMDMs at various times (above lane) after stimulation with IFN-β (250U/ml), IFN-γ (100 ng/ml) or LPS (100 ng/ml). Data are representative of two independent experiments.

Previously generated caspase-1-deficient mouse strains using embryonic stem cells from the 129 strain lack caspase-11 expression^16^. To overcome this confounding factor, we used CRISPR-Cas9 technology and C57BL/6 J embryonic stem cells to generate a caspase-1–deficient mouse strain, which we designate “*Casp1*^Null^” mice (Supplementary Fig. 1). We generated primary bone marrow-derived macrophages (BMDMs) from *Casp1*^Null^ mice and assessed the expression of caspase-11 proteins following stimulation with IFN-β, IFN-γ or LPS. We found inducible expression of caspase-11 in WT and *Casp1*^Null^ BMDMs stimulated with IFN-β, IFN-γ or LPS, whereas *Casp11*^−/−^ BMDMs, as expected, did not (Fig. 1). We also confirmed that caspase-1 expression was absent in *Casp1*^Null^ BMDMs and intact in WT and *Casp11*^−/−^ BMDMs (Fig. 1).

### Inflammasome activities are impaired in *Casp1* ^Null^ BMDMs in response to canonical inflammasome activation

**Figure 2:**
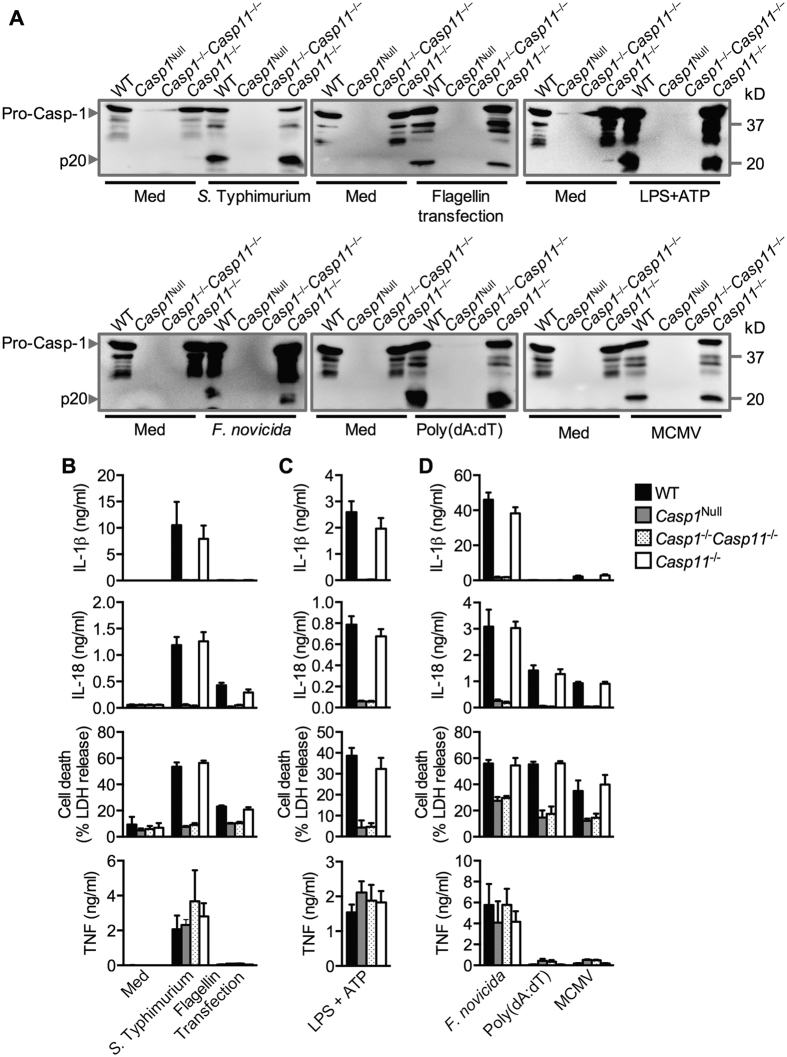
Responses of *Casp1*^Null^ bone marrow-derived macrophages to the activation of canonical inflammasomes. (**A**) Top, immunoblot analysis of pro-caspase-1 (Pro-Casp-1) and the caspase-1 subunit p20 (Casp-1 p20) in unprimed WT or mutant BMDMs left untreated (medium alone [Med]) or assessed 4 h after infection with *Salmonella* Typhimurium (MOI, 1; left) or 4 h after transfection of *S* Typhimurium flagellin (4 μg/ml; middle) or in LPS-primed BMDMs left untreated (Med) or assessed 30 min after stimulation with 5 mM ATP (LPS + ATP, right). Bottom, immunoblot analysis of pro-caspase-1 (Pro-Casp-1) and the caspase-1 subunit p20 (Casp-1 p20) in unprimed WT or mutant BMDMs left uninfected (medium alone [Med]) or assessed 20 h after infection with *F. novicida* (MOI, 100; left) or 5 h after transfection with poly(dA:dT) (5 μg/ml; middle) or 10 h after infection with mouse cytomegalovirus (MCMV, MOI, 10; right). (**B–D**) Release of IL-1β, IL-18, death of BMDMs, and release of TNF after treatment as in (**A**). Cell death indicates % of LDH release relative to total lysis, set at 100% (**B–D**). Data in (**A–D**) are representative of three independent experiments (mean and s.e.m. of values from three independent experiments in **B–D**).

Caspase-1 is activated within an inflammasome following engagement of the inflammasome-initiating sensors NAIP–NLRC4, NLRP3 and AIM2^13^. To systematically validate the *Casp1*^Null^ line, we measured inflammasome responses from primary BMDMs generated from this line following stimulation with known inflammasome triggers. The NAIP–NLRC4 inflammasome can be activated by *Salmonella enterica* serovar Typhimurium (*S.* Typhimurium) which had been grown to a log-phase or through transfection of bacterial flagellin into the host cytoplasm^17^,^18^,^19^,^20^,^21^,^22^. Activation of the NLRC4 inflammasome using *S.* Typhimurium or transfection of flagellin from *S.* Typhimurium led to robust activation of caspase-1, release of IL-1β and/or IL-18, and cell death in WT and *Casp11*^−/−^ BMDMs, but not in *Casp1*^Null^ and *Casp1*^−/−^*Casp11*^−/−^ BMDMs (Fig. 2A and B).

The canonical NLRP3 inflammasome responds to a variety of activators, including ATP, nigericin, bacterial toxin, RNA and crystalline substances^23^,^24^,^25^,^26^. We found that while WT BMDMs secreted IL-1β and IL-18 and underwent cell death in response to LPS and ATP, *Casp1*^Null^ and *Casp1*^−/−^*Casp11*^−/−^ BMDMs failed to do so (Fig. 2A and B).

Activation of the DNA sensor AIM2 by infection with the Gram-negative bacterium *Francisella novicida*, transfection of dsDNA ligand poly(dA:dT) into the cytoplasm or infection by the DNA virus mouse cytomegalovirus (MCMV) leads to assembly of the inflammasome^27^,^28^,^29^,^30^,^31^,^32^,^33^,^34^. Engagement of AIM2 using these microbial and synthetic agents induced maturation of caspase-1, release of IL-1β or IL-18, and cell death in both WT and *Casp11*^−/−^ BMDMs, whereas impaired secretion of IL-1β or IL-18 and cell death were observed in *Casp1*^Null^ and *Casp1*^−/−^*Casp11*^−/−^ BMDMs (Fig. 2A and B). Secretion of the inflammasome-independent cytokine TNF was normal in BMDMs of all genotypes (Fig. 2B).

The Gram-positive bacterium *Listeria monocytogenes* activates caspase-1 via multiple inflammasome-initiating sensors, including the AIM2, NLRP3 and NLRC4^25^,^29^,^35^,^36^,^37^,^38^,^39^,^40^. We found that *Casp1*^Null^ and *Casp1*^−/−^*Casp11*^−/−^ BMDMs failed to secrete IL-1β or IL-18 and undergo cell death (Supplementary Fig. 2A and B). However, we found no role for caspase-11 in the activation of the inflammasome induced by the *L. monocytogenes* (Supplementary Fig. 2A and B)^41^, consistent with the notion that caspase-11 mediates recognition of LPS from Gram-negative bacteria^16^,^42^,^43^,^44^. We have also recently shown that *Casp1*^Null^ mice, similar to *Casp1*^−/−^*Casp11*^−/−^ mice^45^,^46^,^47^,^48^, are more susceptible to infection with *F. novicida* than WT mice, confirming the biological importance of caspase-1 in a mouse model of bacterial infection^49^.

### Secretion of IL-1β and IL-18, but not pyroptosis, is impaired in *Casp1* ^Null^ BMDMs in response to non-canonical inflammasome activation

**Figure 3:**
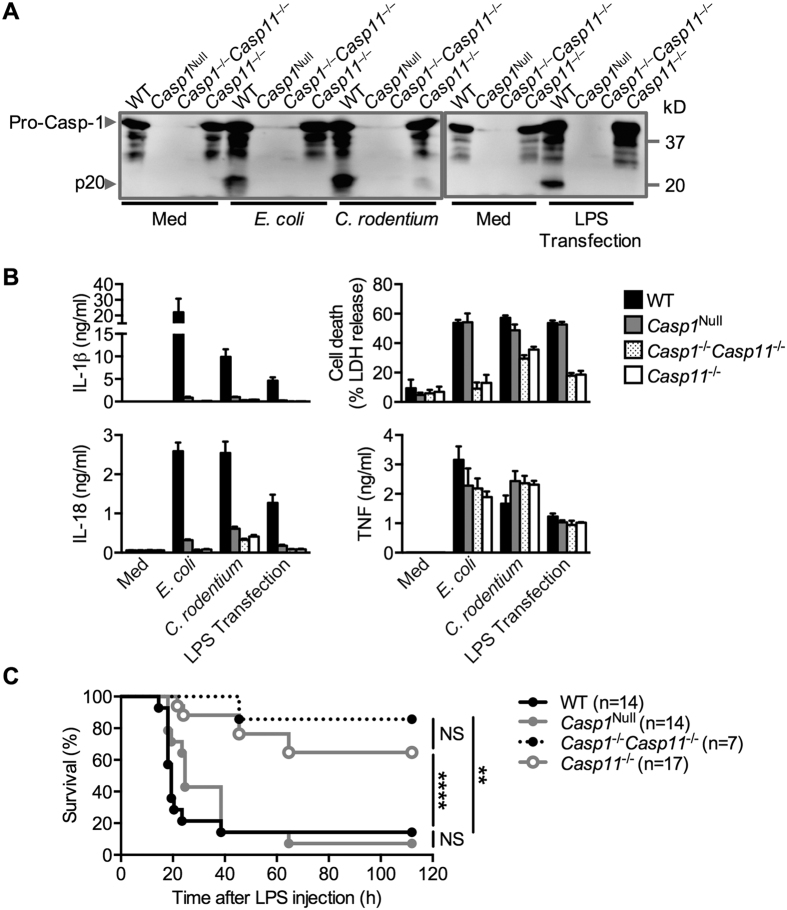
Responses of *Casp1*^Null^ bone marrow-derived macrophages and mice to activation of the non-canonical inflammasome. (**A**) Immunoblot analysis of pro-caspase-1 (Pro-Casp-1) and the caspase-1 subunit p20 (Casp-1 p20) in unprimed WT or mutant BMDMs left untreated (medium alone [Med]) or assessed 20 h after infection with *C. rodentium* (MOI, 20, left), *E. coli* (MOI, 20, middle), or 10 h after LPS transfection (4 μg/ml, right). (**B**) Release of IL-1β, IL-18, death of BMDMs, and release of TNF after treatment as in (**A**). (**C**) Survival of 8-week-old WT and mutant mice injected intraperitoneally with 54 mg LPS per kg body weight. NS, not statistically significant, **P < 0.01 and ****P < 0.0001 (log-rank test). Data are representative of two (**C**) or three independent experiments (**A** and **B**; mean and s.e.m. are representative of values from three independent experiments in **B**).

Gram-negative bacteria, including *Escherichia coli* and *Citrobacter rodentium*, introduce LPS into the host cytoplasm during infection and engage non-canonical activation of the NLRP3 inflammasome via caspase-11^16^. *Casp1*^Null^ BMDMs did not secrete IL-1β and IL-18 in response to infection by *E. coli* and *C. rodentium* or transfection of LPS (Fig. 3A and B). However, cell death was observed in *Casp1*^Null^ BMDMs in response to infection by *E. coli* and *C. rodentium* or transfection of LPS (Fig. 3B), which is consistent with the model that, in response to non-canonical activation of the NLRP3 inflammasome, pyroptosis is driven by caspase-11 rather than caspase-1^16^,^42^,^44^,^50^.

Previous studies have demonstrated that *Casp1*^−/−^*Casp11*^−/−^ mice are resistant to acute LPS endotoxemia^15^,^16^,^51^, suggesting that caspase-1 and/or caspase-11 mediate LPS-induced lethality. Further studies have shown that *Casp11*^−/−^ mice are resistant to acute LPS endotoxemia^42^,^44^,^52^,^53^, arguing that caspase-11 is a main driver of LPS-induced lethality. To investigate the function of caspase-1 in this model, Kayagaki and colleagues microinjected a bacterial artificial chromosome transgene encoding caspase-11 into *Casp1*^−/−^*Casp11*^−/−^ mouse embryos, re-establishing caspase-11 expression in this mouse line (referred to as the *Casp1*^−/−^*Casp11*^Tg^ mouse strain)^16^. Using this mouse strain, they reported that *Casp1*^−/−^*Casp11*^Tg^ mice were modestly resistant to LPS-induced lethality compared with WT mice^16^.

To investigate the contribution of caspase-1 in LPS endotoxemia in *Casp1*^Null^ mouse strain, we injected LPS into WT, *Casp1*^Null^, *Casp1*^−/−^*Casp11*^−/−^ and *Casp11*^−/−^ mice and monitored their survival. We found that 86% of WT (12/14) and 93% *Casp1*^Null^ (13/14) mice succumbed to LPS-induced endotoxemia (not statistically significant, *P* = 0.031) (Fig. 3C). *Casp1*^Null^ mice succumbed with a slightly delayed kinetic compared with WT mice, consistent with previous observations^16^. In contrast, only 35% of the *Casp11*^−/−^ mice (6/17, *P* < 0.0001 compared with WT) and 14% *Casp1*^−/−^*Casp11*^−/−^ (1/7, *P* = 0.0014 compared with WT) mice succumbed to endotoxemia (Fig. 3C). These data suggested a dominant role of caspase-11 and a minor role of caspase-1 in mediating acute lethal endotoxemia. Taken together, we have validated our *Casp1*^Null^ mice for use in the study of inflammasome biology.

### Differential roles of caspase-1 and caspase-11 in response to infection with the fungal pathogen *Aspergillus fumigatus*

**Figure 4:**
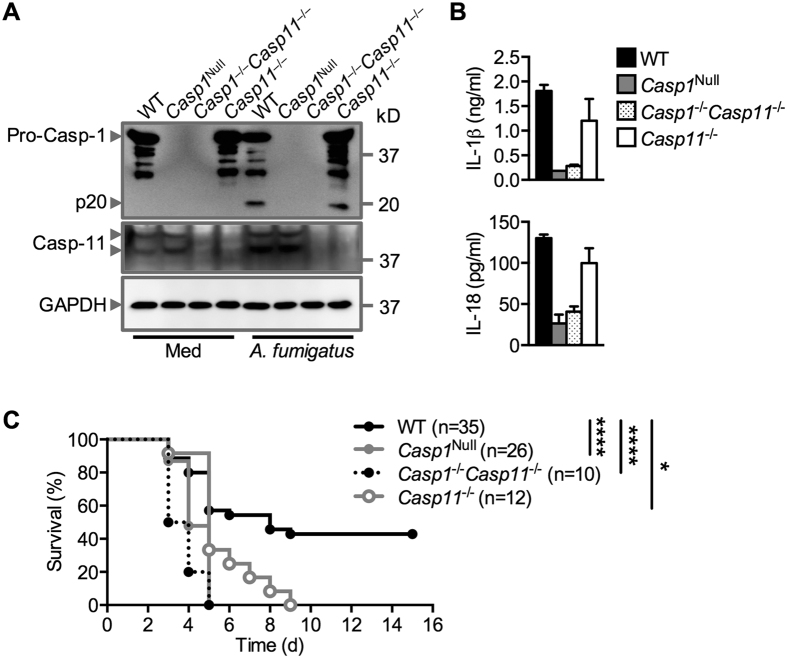
Differential roles of caspase-1 and caspase-11 in response to infection with *Aspergillus fumigatus*. (**A**) Immunoblot analysis of pro-caspase-1 (Pro-Casp-1) and the caspase-1 subunit p20 (Casp-1 p20) and GAPDH (loading control) in unprimed WT or mutant bone marrow-derived dendritic cells left untreated (medium alone [Med]) or assessed 20 h after infection with *A. fumigatus* (MOI, 10). (**B**) Release of IL-1β and IL-18 after treatment as in (**A**). (**C**) Survival of 8-week-old WT and mutant mice infected with 5 × 10^5^ *A. fumigatus* conidia after immunosuppression with cyclophosphamide and cortisone acetate. *P < 0.05, ****P < 0.0001 (log-rank test). Data are representative of two (**C**) or three independent experiments (**A** and **B**; mean and s.e.m. are representative of values from three independent experiments in **B**).

In addition to its function in the recognition of bacteria and viruses, inflammasomes have a central role in the control of fungal pathogens, including *Aspergillus fumigatus*^54^. We found that *A. fumigatus* failed to induce the release of IL-1β and IL-18 in *Casp1*^Null^ and *Casp1*^−/−^*Casp11*^−/−^ bone marrow-derived dendritic cells (BMDCs), whereas maturation of caspase-1 and the release of IL-1β and IL-18 in WT and *Casp11*^−/−^ BMDCs were observed (Fig. 4A and B). This finding supported our previous observations showing that caspase-11 is dispensable for activation of the inflammasome induced by *A. fumigatus* infection^55^. Similar to BMDMs, *Casp1*^Null^ BMDCs retained the ability to express the caspase-11 protein (Fig. 4A).

We have previously found that *Casp1*^−/−^*Casp11*^−/−^ mice were extremely sensitive to infection by *A. fumigatus* compared with WT mice^55^. However, whether caspase-1 or caspase-11 contributed to the host defense against *A. fumigatus* infection *in vivo* has remained unclear. To investigate this, we immunocompromised WT, *Casp1*^Null^, *Casp1*^−/−^*Casp11*^−/−^ and *Casp11*^−/−^ mice with cyclophosphamide and cortisone acetate and intranasally infected these mice with *A. fumigatus* conidia. Immunosuppression procedures were used because immunocompetent WT mice and mice lacking components of the inflammasome do not succumb to infection with *A. fumigatus*^55^, which is in line with the observation that only immunocompromised individuals in large are susceptible to invasive pulmonary aspergillosis^56^.

Following intranasal infection with *A. fumigatus* conidia, *Casp1*^Null^ mice were substantially more susceptible to *A. fumigatus*–induced mortality compared with WT mice (Fig. 4C). The hypersusceptibility of *Casp1*^Null^ mice to *A. fumigatus* was phenocopied by *Casp1*^−/−^*Casp11*^−/−^ mice (Fig. 4C). In addition, we found that *Casp11*^−/−^ mice were also more susceptible to infection with *A. fumigatus* conidia compared with WT mice (Fig. 4C). However, *Casp11*^−/−^ mice succumbed to infection with a delayed kinetic compared with *Casp1*^Null^ mice or *Casp1*^−/−^*Casp11*^−/−^ mice. Although caspase-11 had no role in the activation of the inflammasome in BMDCs in response to *A. fumigatus,* caspase-11 contributed to the host defense against *A. fumigatus* infection *in vivo*. It is possible that activation of caspase-11 might induce pyroptosis and/or actin-mediated phagosomal killing in a cell-type-specific manner in order to control *A. fumigatus* dissemination *in vivo*^57^,^58^,^59^. Indeed, the release of IL-18 via *A. fumigatus*-sensing NLRP3 and AIM2 inflammasomes induces production of IFN-γ, which might provide a priming signal for caspase-11 to clear *A. fumigatus in vivo*^55^. This IL-18–IFN-γ–Caspase-11 signaling pathway and defense strategy has been reported in the host clearance of the cytosolic bacterium *Burkholderia thailandensis*^60^. Overall, our study has generated and validated a valuable genetic tool to enable us to refine the differential contribution of caspase-1 and capsase-11 in health and disease in future studies.

## Discussion

Inflammatory caspases are multi-functional proteins which mediate host defense to infectious diseases and regulate tumor development, metabolic syndromes, autoinflammatory disease, tissue repair, and cell survival^1^,^2^. Previously generated caspase-1–deficient mouse strains using embryonic stem cells of the 129 background lack caspase-11 expression, essentially rendering them deficient in both caspase-1 and caspase-11^16^. Therefore, the biological insights of caspase-1 gained from using *Casp1*^−/−^*Casp11*^−/−^ mice should be re-examined. We generated caspase-1–deficient mouse strain on the C57BL/6 J background to provide the scientific community a genetic tool to revisit the biological functions of caspase-1 and caspase-11.

Studies into the molecular mechanisms regulating caspase-1 and caspase-11 have revealed important differences between these proteases. Mouse caspase-1 shares 46% amino acid sequence identity with mouse caspase-11^61^. Caspase-1 is unequivocally required for the proteolytic processing of pro-IL-1β and pro-IL-18 and for pyroptosis in response to canonical inflammasome activators^2^,^3^,^4^,^5^,^8^,^9^,^10^. Caspase-11 cannot directly proteolytically process pro-IL-1β and pro-IL-18^61^, although previous studies suggest that one of the caspase-11 homologs in humans, caspase-4, could cleave pro-IL-1β and pro-IL-18^62^,^63^. Caspase-11 itself is capable of driving pyroptosis in a caspase-1–independent manner in response to non-canonical activation of the inflammasome, that is, in response to transfection of LPS or during infection by certain Gram-negative bacteria, including *C. rodentium, E. coli* and *Vibrio cholerae*^16^. In this case, expression of caspase-11 is mediated by interferon signaling following TLR4–dependent recognition of extracellular LPS from most Gram-negative bacteria^64^,^65^,^66^,^67^,^68^. Caspase-11 recognizes LPS introduced into the host cytoplasm^16^,^42^,^43^,^44^, which induces caspase-11–dependent cleavage of the pro-pyroptotic factor gasdermin D^69^,^70^,^71^. The N-terminal fragment of gasdermin D mediates pore formation on the cell membrane that leads to pyroptosis and activation of caspase-1 via the NLRP3 inflammasome^69^,^70^,^71^,^72^,^73^,^74^,^75^,^76^. However, direct interaction between caspase-11 and caspase-1 resulting in the activation of caspase-1 has also been reported^52^,^61^,^77^. A more recent study also demonstrated that caspase-11 can be activated by host-derived oxidized phospholipids in dendritic cells to drive IL-1β release without pyroptosis^78^. In our study, the residual cell death observed in *Casp1*^Null^ BMDMs in response to *F. novicida* or *Casp11*^−/−^ BMDMs in response to *C. rodentium* might indicate a minor contribution from inflammasome-independent cell death pathways. *F. novicida* can engage apoptosis via a caspase-1-independent, caspase-8-dependent mechanism^79^,^80^,^81^. *C. rodentium* or its relative enteropathogenic *E. coli* and enterohemorrhagic *E. coli* encode the effector proteins NleB^82^,^83^ and NleH^84^ to actively suppress apoptosis and/or necroptosis. These data suggest that downregulation of NleB and NleH over the course of infection by these enteropathogens might engage cell death pathways other than pyroptosis.

Studies in mouse models have revealed differential contributions between caspase-1 and caspase-11 in infection and cancer. *Casp1*^−/−^*Casp11*^−/−^ mice are hypersusceptible to infection by *S.* Typhimurium 18,65,85,86. A further study has found that the caspase-11-expressing mouse strain *Casp1*^−/−^*Casp11*^Tg^ (generated via microinjection of a bacterial artificial chromosome transgene encoding caspase-11 into *Casp1*^−/−^*Casp11*^−/−^ mouse embryos) revealed that they were more susceptible to infection with *S.* Typhimurium than *Casp1*^−/−^*Casp11*^−/−^ mice^65^. However, this study reported that *Casp11*^−/−^ mice were not more susceptible compared with wild-type mice^65^. These data would suggest that caspase-11 is detrimental to the host only in the absence of caspase-1. However, others have reported a protective role for caspase-11 during salmonellosis exclusively in the intestine^63^,^87^. In mouse macrophages or embryonic fibroblasts, both caspase-1 and caspase-11 contribute to cell-autonomous control of intracellular replication of *S.* Typhimurium and other bacteria^21^,^58^,^88^,^89^,^90^.

In the context of intestinal inflammation, *Casp1*^−/−^*Casp11*^−/−^ mice are susceptible to colitis induced by the colitogen dextran sulfate sodium (DSS)^91^,^92^,^93^. More recent studies have now revealed that mice lacking caspase-11 alone are sensitive to DSS-induced colitis^94^,^95^,^96^. The susceptibility observed in *Casp11*^−/−^ mice largely phenocopies that of *Casp1*^−/−^*Casp11*^−/−^ mice^94^, suggesting that caspase-11 could be a dominant inflammatory caspase that drives a protective response in the intestine. This protective response in the intestine might be attributed to the ability of caspase-11 to mediate secretion of IL-18^95^,^96^, a cytokine generally considered to be protective in colitis. Caspase-1 could have non-redundant functions with caspase-11 in the intestine and that further studies are required to investigate their relative effect during intestinal inflammation.

The caspase-11 homologs in humans, caspase-4 and caspase-5, also recognize LPS and activate caspase-1, mediate cleavage of gasdermin D, and drive pyroptosis^43^,^69^,^70^. However, subtle differences have been reported between human caspase-4 and human caspase-5. Caspase-4, but not caspase-5, is required for cell death and IL-1β production in the human monocytic THP-1 cell line following transfection of LPS^97^,^98^. Caspase-4 also mediates secretion of IL-1β and IL-18 in LPS-stimulated mouse BMDMs engineered to express human caspase-4^99^. However, other studies have suggested that both caspase-4 and caspase-5 are required for IL-1β release in human monocytes stimulated with LPS or in the THP-1 cell line infected with *S.* Typhimurium^97^,^100^, suggesting non-redundant activities between these caspases. A further study has demonstrated that caspase-4 is not necessary for IL-1β release in primary human macrophages infected with *S.* Typhimurium, *L. pneumophila and Y. pseudotuberculosis*^101^. The function of these inflammatory caspases is likely to be cell-type- and species-specific and influenced by the type of activators delivered into the cell.

Overall, our study has generated and validated a mouse strain for use in unraveling the specific function of caspase-1 without the confounding absence of caspase-11. We also showed the unique and overlapping functions of caspase-1 and caspase-11 during infection and inflammation. Two recent studies have now reported caspase-1-deficient mice on the C57BL/6 background^102^,^103^. Together with our study, the availability of caspase-1-deficient mice provides a valuable tool for the scientific community to propel investigations that aim to refine the differential functions of caspase-1 and caspase-11 in health and disease.

## Methods

### Mice

*Casp1*^−/−^*Casp11*^−/−^ (also known as *Casp1*^−/−^*Casp11*^129mt*/*129mt^) and *Casp11*^−/−^ mice have been described previously^16^. WT C57BL/6 J mice were purchased from the Jackson Laboratory and bred at St. Jude Children’s Research Hospital. Animal studies were conducted under protocols approved by the St. Jude Children’s Research Hospital on the Use and Care of Animals.

### Generation of *Casp1* ^Null^ mice

Pronuclear-stage C57BL/6 J zygotes were injected with 2 single guide RNAs (sgRNAs) (Casp1-Guide-01: ATTCTTGACGTCTTAATCTC [125 ng/μL] and Casp1-Guide-02: TTGGGACATTGCAACGAACT [125 ng/μL]) designed to introduce DNA double strand breaks into intron 1 and intron 4 of the *Casp1* gene, and a human codon optimized Cas9 mRNA transcript (50 ng/μL) (Fig. S1), and were subsequently surgically transplanted into the oviducts of pseudo pregnant CD1 females. Newborn mice bearing a null allele of *Casp1 (Casp1*^Null^) were identified by amplification of a 716 bp fragment using primers flanking the 2 break sites [Casp1-F51 and Casp1-R32 (Tables S1 and S2)]. Sanger sequencing of the 716 bp amplicon confirmed proper deletion of the ~3.8 kb fragment containing exons 2–4. sgRNAs were designed and generated as described previously^104^. The Cas9 mRNA transcript was generated as described previously^104^. Potential off-target sites were identified using Cas-OFFinder and each locus was PCR-amplified and sequenced (Table S3)^105^. No off-target site cleavage was observed.

### PCR genotyping

Genotyping of the *Casp1* locus was performed using primers flanking each sgRNA target site using the following primer pairs (Fig. S1, and Tables S1 and S2): Casp1-F51 and Casp1-R52 (5′ target site), Casp1-F31 and Casp1-R32 (3′ target site), and Casp1-F51 and Casp1-R32 (*Casp1* allele).

### Microbial culture

*S.* Typhimurium SL1344, *Citrobacter rodentium* (51459, American Type Culture Collection) and *Escherichia coli* (11775, American Type Culture Collection) were inoculated into Luria-Bertani media (3002–031, MP Biomedicals) and incubated under aerobic conditions overnight at 37 °C. *S.* Typhimurium SL1344 was subcultured (1:10) into fresh LB media for 3 h at 37 °C to generate log-phase grown bacteria. *F. novicida* strain U112 were grown in BBL™ Trypticase™ Soy Broth (TSB) (211768, BD) supplemented with 0.2% L-cysteine (BP376-100, ThermoFisher Scientific) under aerobic conditions overnight at 37 °C. *F. novicida* was subcultured (1:10) in fresh TSB supplemented with 0.2% L-cysteine for 4 h and resuspended in PBS. *L. monocytogenes* was cultured in brain heart infusion broth (211059, BD) overnight. *Aspergillus fumigatus* CBS144-89 was grown on 2% (wt/vol) malt− 2% (wt/vol) agar slants for 1 week at room temperature, and conidia were harvested in water containing 0.05% (vol/vol) Tween 80^106^.

### Cultivation and stimulation of bone marrow-derived macrophages and dendritic cells

BMDMs and BMDCs were cultured as described previously^46^,^55^. In brief, BMDMs were generated from mouse bone marrow cells grown after 5–6 days in DMEM (11995073, ThermoFisher Scientific) supplemented with 10% FBS (TMS-013-B, Millipore), 30% L929 conditioned media and 1% penicillin and streptomycin (15070-063, ThermoFisher Scientific). BMDMs were seeded in antibiotic–free media at a concentration of 1 × 10^6^ cells onto 12-well plates and incubated overnight. BMDCs were generated from mouse bone marrow cells grown over 7 days in RPMI 1640 (10-040-CV, Corning) supplemented with 10% FBS, 1% penicillin-streptomycin, 1% non-essential amino acid (11140, ThermoFisher Scientific), 1% sodium pyruvate (11360, ThermoFisher Scientific), and 20 ng/ml GM-CSF.

The following conditions were used to stimulate BMDMs: *F. novicida* (MOI 100 and 20 h for caspase-1 activation), *S.* Typhimurium (MOI 1, 4 h), *C. rodentium* (MOI 20 for 20 h), *E. coli* (MOI 20 for 20 h) and *L. monocytogenes* (MOI 20, 8 h). 50 μg/ml gentamicin (15750-060, ThermoFisher Scientific) was added after 2 h (*S.* Typhimurium), 4 h (*C. rodentium, E. coli* and *L. monocytogenes*), and 8 h (*F. novicida*) post-infection to kill extracellular bacteria. To activate the canonical NLRP3 inflammasome, BMDMs were primed using 500 ng/ml ultrapure LPS from *Salmonella minnesota* R595 (tlrl-smlps, InvivoGen) for 4 h and stimulated with 5 mM ATP (10127531001, Roche) for 45 min. The MCMV Smith MSGV strain (VR-1399^™^, American Type Culture Collection) was obtained from P.G. Thomas (St. Jude Children’s Research Hospital). MCMV was added to unprimed BMDMs at an MOI of 10 for 10 h. For DNA transfection, 2.5 μg of poly(dA:dT) (tlrl-patn, InvivoGen) were resuspended in PBS and mixed with 0.3 μl of Xfect polymer in Xfect reaction buffer (631318, Clontech Laboratories, Inc.) per reaction. After 10 min, 50 μl of the DNA–Xfect complex was added to BMDMs in 500 μl Opti-MEM (31985-070, ThermoFisher Scientific) and incubated for 5 h. For LPS transfection, 2 μg of ultrapure LPS from *Salmonella minnesota* R595 (tlrl-smlps, InvivoGen) was resuspended in PBS and mixed with 0.3 μl of Xfect polymer in Xfect reaction buffer per reaction. After 10 min, 50 μl of the LPS–Xfect complex was added to BMDMs in 500 μl Opti-MEM and incubated for 6 h. For flagellin transfection, 2 μg of ultrapure flagellin from *S.* Typhimurium (tlrl-epstfla-5, InvivoGen) was resuspended in PBS and mixed with 20 μl of DOTAP (D1163, Sigma) per reaction. The reaction mixture was incubated for 20 min and added to BMDMs in 500 μl Hank’s Balanced Salt Solution (SH30031.02, HyClone, GE Healthcare Life Sciences). BMDCs were infected with *A. fumigatus* conidia (MOI, 10) for 20 h.

### Lactate dehydrogenase assay

Levels of lactate dehydrogenase released by cells were determined using the CytoTox 96 Non-Radioactive Cytotoxicity Assay according to the manufacturer’s instructions (G1780, Promega). Cell culture supernatants were collected for ELISA.

### Immunoblotting analysis

Cells and supernatant were lysed in RIPA buffer and sample loading buffer containing SDS and 100 mM DTT. Proteins were separated on 8–12% polyacrylamide gels. Following electrophoretic transfer of protein onto PVDF membranes (IPVH00010, Millipore), membranes were blocked in 5% skim milk and incubated with primary antibodies against caspase-1 (1:3,000 dilution, AG-20B-0042, Adipogen), caspase-11 (1:1,000 dilution, NB120-10454, Novus) and GAPDH (1:10,000 dilution, #5174, Cell Signaling Technologies). Membranes were then incubated with HRP-conjugated secondary antibody for 1 h and proteins were visualized using Super Signal Femto substrate (34096, ThermoFisher Scientific).

### Cytokine analysis

Cytokine levels were determined using a multiplex ELISA (MCYTOMAG-70K, Millipore) or IL-18 ELISA (BMS618/3TEN, Affymetrix eBioscience) according to the manufacturers’ instructions.

### LPS-induced endotoxemia

Male or female mice were injected intraperitoneally with 54 mg per kg body weight of LPS (L2630, Sigma) and monitored throughout the day for 5 days.

### *A. fumigatus* infection *in vivo*

Cyclophosphamide monohydrate (C0768, Sigma) was dissolved in sterile PBS and given by intraperitoneal injection (150 mg per kg of body weight). Cortisone 21-acetate (C3130, Sigma) was suspended in 0.05% Tween 80 in PBS and administered by subcutaneous injection (112 mg per kg of body weight). Mice were given a combination of cyclophosphamide and cortisone acetate 2 day prior to infection and the day of infection. Mice were anesthetized by isoflurane inhalation and inoculated intranasally with 5 × 10^5^ conidia from *A. fumigatus* strain CBS144.89 in 30 μl of 0.05% Tween 80 in PBS.

### Statistical analysis

GraphPad Prism 6.0 software was used for data analysis. Data are shown as mean ± s.e.m. Statistical significance was determined by a log-rank test. *P < *0.05 was considered statistically significant.

## Additional Information

**How to cite this article:** Ming Man, S. *et al*. Differential roles of caspase-1 and caspase-11 in infection and inflammation. *Sci. Rep.* **7**, 45126; doi: 10.1038/srep45126 (2017).

**Publisher's note:** Springer Nature remains neutral with regard to jurisdictional claims in published maps and institutional affiliations.

## Supplementary Material

Supplementary Figures and Tables
