# Supplementary material for: Differential roles of caspase-1 and caspase-11 in infection and inflammation
Source: Sci Rep. 2017 Mar 27;7:45126. doi: 10.1038/srep45126 (PMC5366862; doi:10.1038/srep45126)
Supplement: Supplementary Figures and Tables [file srep45126-s1.pdf]

SUPPLEMENTARY INFORMATION

Differential roles of caspase-1 and caspase-11 in infection and inflammation

Si Ming Man, Rajendra Karki, Benoit Briard, Amanda Burton, Sebastien Gingras, Stephane Pelletier & Thirumala-Devi Kanneganti*

Department of Immunology, St. Jude Children's Research Hospital, Memphis, TN, USA.

* Correspondence to:

Thirumala-Devi Kanneganti

Department of Immunology, St. Jude Children's Research Hospital

MS #351, 262 Danny Thomas Place

Memphis TN 38105-3678

Tel: (901) 595-3634

Fax. (901) 595-5766

E-mail: Thirumala-Devi.Kanneganti@STJUDE.ORG

Keywords: Caspase-1, Caspase-11, inflammasome, pyroptosis, IL-1β and IL-18

Supplementary figure legends

Supplementary Figure S1. Generation of *Casp1*^{Null} mice.

Targeting strategy used to inactivate the mouse *Casp1* gene. 2 sgRNAs were designed to target intron 1 and intron 4 of the *Casp1* gene. sgRNAs and the Cas9 mRNA transcript were injected into the C57BL/6J pronuclear-stage zygotes to induce 2 DNA DSBs. Resolution of the DSBs by NHEJ resulted in the generation of mice with several alleles, including mice with a deletion of exons 2 to 4 (*Casp1*$^{-}$). Grey boxes represent exons (numbered). Target regions of Casp1-Guide 1 and Casp1-Guide 2 are shown. Black triangles indicate the cleavage site of the 2 sgRNAs. Splicing of exon 1 into exon 5 induces a frame shift and premature stop codon (red boxes) in Exon 5. Colored arrows illustrate the primers used for genotyping: red arrow, Casp1-F51; yellow arrow, Casp1-R52; blue arrow: Casp1-F31; and green arrow, Casp1-R52.

Supplementary Figure S2. Responses of *Casp1*^{Null} bone marrow-derived macrophages to infection with *Listeria monocytogenes*.

(A) Immunoblot analysis of pro-caspase-1 (Pro-Casp-1) and the caspase-1 subunit p20 (Casp-1 p20) and GAPDH (loading control) in unprimed WT or mutant BMDMs left untreated (medium alone [Med]) or assessed 8 h after infection with *L. monocytogenes* (MOI, 20). (B) Release of IL-1β, IL-18, death of BMDMs, and release of TNF after treatment as in (A). Data are representative of three independent experiments (mean and s.e.m. in B).

Supplementary table legends

Supplementary Table S1. PCR genotyping primers.

Supplementary Table S2. Size of PCR amplicons for each primer pairs and genotype.

Supplementary Table S3. Off-target (OT) sites associated with each sgRNA.

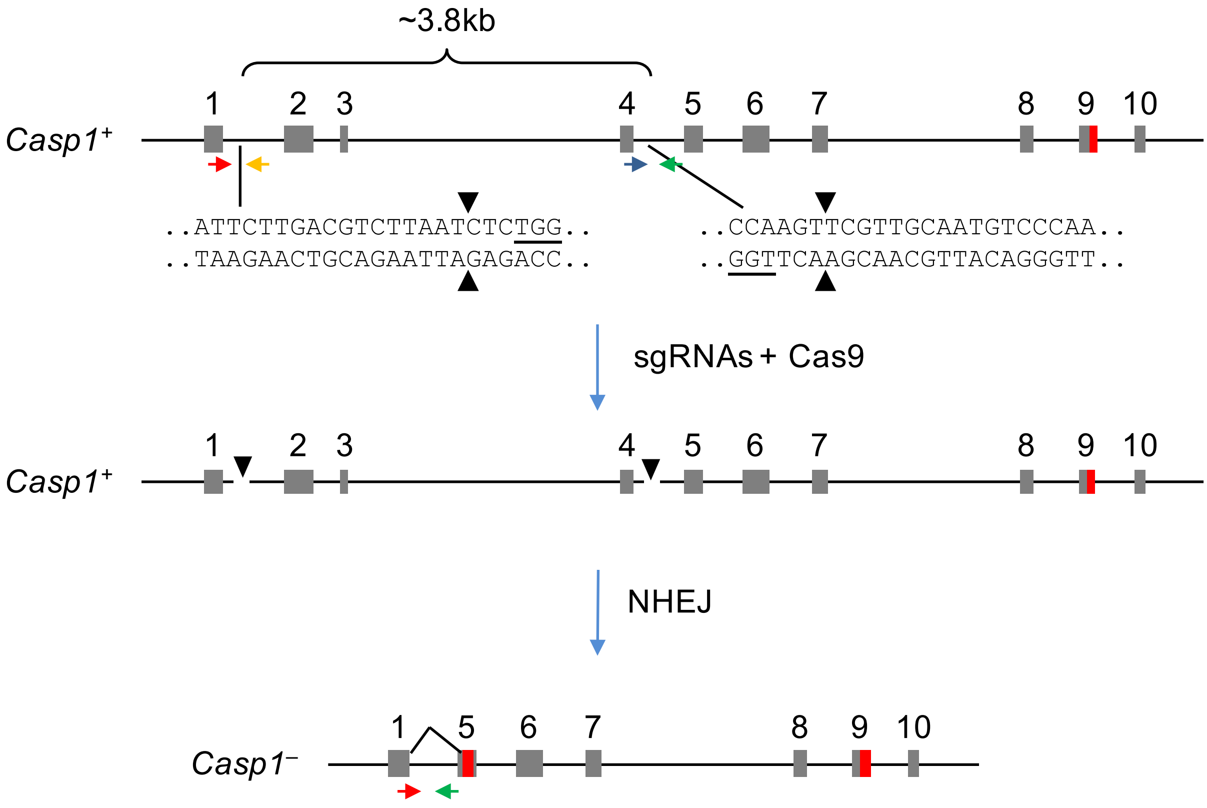

Supplementary Figure S1

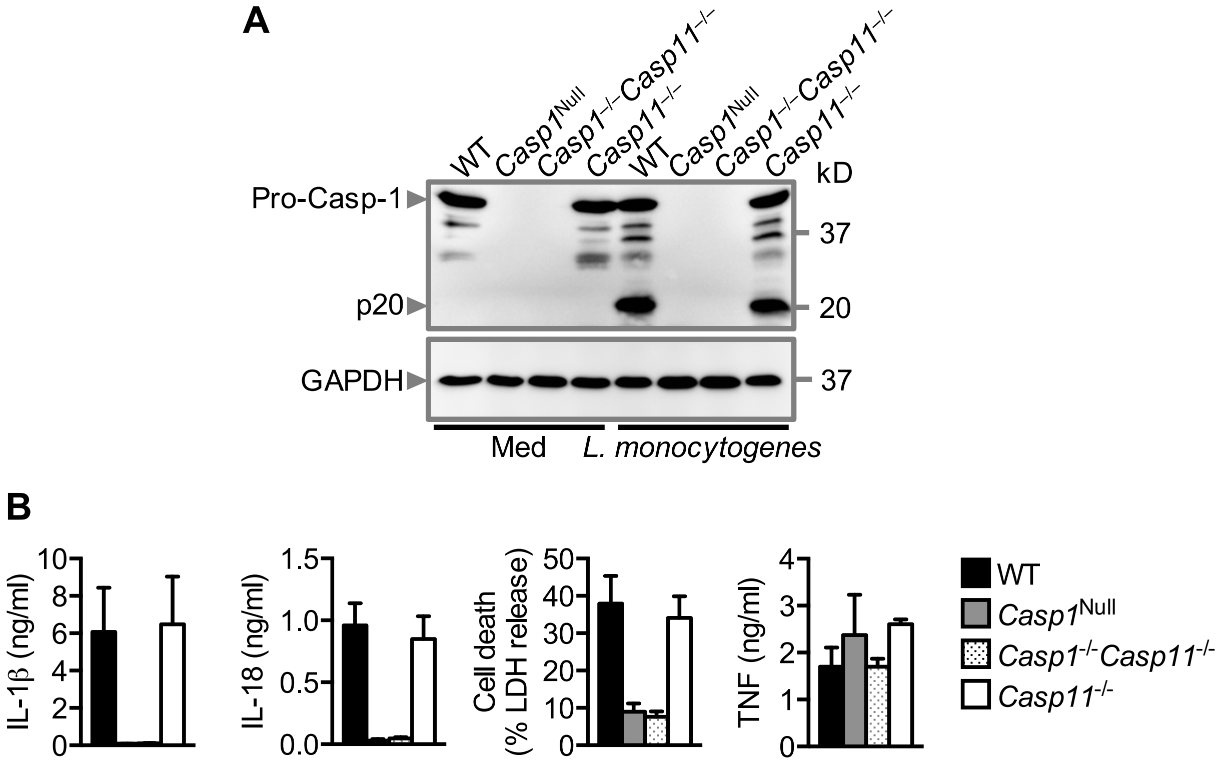

Supplementary Figure S2

Supplementary Table S1. PCR genotyping primers.

Primer	Sequence (5’- to -3’)
Casp1-F51	CGC CCT GTT GGA AAG GAA CT
Casp1-R52	TGC CCT CAG GAT CTT GTC TG
Casp1-F31	GTA TTG AGC CCA TGG TTG CC
Casp1-R32	AGC TCC AAC CCT CGG AGA AA

Supplementary Table S2. Size of PCR amplicons for each primer pairs and genotype.

Primer pair	PCR amplicon size (bp)	
	Casp1^{+}	*Casp1*^{Null}
Casp1-F51 + Casp1-R52 (5' locus)	650	N/A
Casp1-F31 + Casp1-R32 (3' locus)	786	N/A
Casp1-F51 + Casp1-R32 (*Casp1* allele)	N/A	716

Supplementary Table S3. Off-target (OT) sites associated with each sgRNA.

Off-target	chr	Position	Off-target sequence	Strand	# mm
Casp1_guide 1_OT1	chr8	106262966	ATTCTTGACaTCTTAcTCcCGAG	-	3
Casp1_guide 1_OT2	chr4	72514035	ATTCTTGACtgCTTAcTCTCCAG	-	3
Casp1_guide 1_OT3	chr19	29328947	ATTCTTGAaGaCTaAATCTCCAG	-	3
Casp1_guide 1_OT4	chr19	45270468	ATgCTTGACcTCTTAAaCTCAAG	+	3
Casp1_guide 1_OT5	chr15	27186058	ATTCTTGAgGTCcTgATCTCAAG	+	3
Casp1_guide 1_OT6	chr17	62305294	ATTtTTGACaTCTTAATCTaAAG	+	3
Casp1_guide 1_OT7	chr10	44565955	ATTCTTcAtGTtTTAATCTCTAG	-	3
Casp1_guide 1_OT8	chr10	92440424	AaTCTTGAtGTCTTAtTCTCCAG	-	3
Casp1_guide 1_OT9	chr10	112267086	ATTCTgGACtTCTTAgTCTCCGG	+	3
Casp1_guide 1_OT10	chrX	98348681	ATTCTTGAaGTaTTAAaCTCAAG	+	3
Casp1_guide 1_OT11	chrX	116810172	ATTCTTGgtGcCTTAATCTCTGG	-	3
Casp1_guide 1_OT12	chr18	44922066	ATTCTTGACtTCaTAAcCTCGAG	+	3
Casp1_guide 2_OT1	chr8	125177122	TTGGGACATTtCAgCaAACTAAG	-	3
Casp1_guide 2_OT2	chr3	78902094	TTGGGACAcTGCAtgGAACTGGG	-	3
Casp1_guide 2_OT3	chr5	118614890	TTGGGAgATTGtAAgGAACTGGG	+	3
Casp1_guide 2_OT4	chr16	14975342	TTGGGcCAcTGCAAaGAACTGGG	-	3
Casp1_guide 2_OT5	chr16	37595704	TTGGatCATTGCtACGAACTGAG	-	3
Casp1_guide 2_OT6	chr1	130527014	TTtGGACATTcCAACaAACTGAG	+	3
Casp1_guide 2_OT7	chr1	130525775	TTtGGACATTcCAACaAACTGAG	-	3
Casp1_guide 2_OT8	chr13	32642148	TTGGaACATaGCAAaGAACTTAG	+	3
Casp1_guide 2_OT9	chr10	119424402	TTGGGACATTGCccaGAACTCAG	+	3
Casp1_guide 2_OT10	chr10	127802515	TTGGGACAaTGgAACGAgCTGAG	-	3
Casp1_guide 2_OT11	chrX	94533053	TgtGGACATTGCcACGAACTAAG	+	3
